# Human Milk’s Hidden Gift: Implications of the Milk Microbiome for Preterm Infants’ Health

**DOI:** 10.3390/nu11122944

**Published:** 2019-12-04

**Authors:** Isadora Beghetti, Elena Biagi, Silvia Martini, Patrizia Brigidi, Luigi Corvaglia, Arianna Aceti

**Affiliations:** 1 Neonatal Intensive Care Unit, AOU Bologna, Department of Medical and Surgical Sciences (DIMEC), University of Bologna. via Massarenti, 11-40138 Bologna, Italy; i.beghetti@gmail.com (I.B.); silvia.martini4@gmail.com (S.M.); luigi.corvaglia@unibo.it (L.C.); 2 Unit of Molecular Ecology of Health, Department of Pharmacy and Biotechnology (FABIT), University of Bologna. Via Belmeloro, 6–40126 Bologna, Italy; elena.biagi@unibo.it (E.B.); patrizia.brigidi@unibo.it (P.B.)

**Keywords:** human milk microbiota, preterm infants, breastfeeding, donor human milk

## Abstract

Breastfeeding is considered the gold standard for infants’ nutrition, as mother’s own milk (MOM) provides nutritional and bioactive factors functional to optimal development. Early life microbiome is one of the main contributors to short and long-term infant health status, with the gut microbiota (GM) being the most studied ecosystem. Some human milk (HM) bioactive factors, such as HM prebiotic carbohydrates that select for beneficial bacteria, and the specific human milk microbiota (HMM) are emerging as early mediators in the relationship between the development of GM in early life and clinical outcomes. The beneficial role of HM becomes even more crucial for preterm infants, who are exposed to significant risks of severe infection in early life as well as to adverse short and long-term outcomes. When MOM is unavailable or insufficient, donor human milk (DHM) constitutes the optimal nutritional choice. However, little is known about the specific effect of DHM on preterm GM and its potential functional implication on HMM. The purpose of this narrative review is to summarize recent findings on HMM origin and composition and discuss the role of HMM on infant health and development, with a specific focus on preterm infants.

## 1. Introduction

Nutrition in early life plays a key role in shaping an infant’s future health. Specifically, human milk (HM) is known to exert a series of beneficial effects for infants, including improved neurological, immunological, and metabolic outcomes. Breastfeeding is considered the gold standard for infants’ nutrition, as mother’s own milk (MOM) provides all the nutritional factors required for optimal infant development. In addition to its nutritional content, MOM constitutes “nature’s first functional food” [1], as it harbours a variety of bioactive factors, including soluble immune factors, antimicrobial proteins and peptides, functional fatty acids, hormones, oligosaccharides, nucleic acids, stem cells, antioxidants, and a wide array of microbes known as the HM microbiome (HMM) [2,3,4,5,6,7]. The ongoing acknowledgment of the role of the microbiome in human health and disease had led to an increased scientific interest for HMM. Early life microbiome is now recognized as one of the main contributors to short and long-term infant health status [8], and included among key participants in the Developmental Origins of Health and Disease (DOHaD) [9]. According to the DOHaD hypothesis, environmental exposures during critical time windows in early life can alter foetal and infant programming, thus leading to alterations in health status [10]. As for the microbiome assembly, transient variations in the composition and function of human microbiome in this critical developmental window have been associated with several non-communicable diseases in infancy and childhood, such as necrotizing enterocolitis (NEC), asthma and atopic disease, obesity, and neurodevelopmental disorders [9].

The gut microbiota (GM) constitutes the most studied ecosystem in infants: Many factors, such as gestational age, mode of delivery, antibiotics, type of nutrition, and social environment, impact on the composition of the infant’ early GM. Among these factors, some HM bioactive factors, such as HM prebiotic non-digestible carbohydrates (i.e., human milk oligo-saccharides (HMOs)) that select for beneficial bacteria, and the specific HM microbiota, are emerging as early mediators in the relationship between the development of GM in early life and short and long-term health outcomes. 

In this review we will summarize recent findings on HMM origin and composition and discuss the role of HMM on infant health and development, with a specific focus on preterm infants.

## 2. The Human Milk Microbiome

Until recently, HM was considered a sterile fluid. Although there existed reports of viable bacteria in HM from healthy women, these bacteria were thought to be contaminants from the skin or other environmental sources [11]. The long-standing dogma of HM sterility was so well-established that the large Human Microbiome Project [12], which was aimed at describing the microbiome features from multiple body sites, did not include HM or the mammary gland among the sites of interest. Nonetheless, most experts now agree about the existence of a specific HMM [13], which is responsible for the recent definition of HM as “mother nature’s prototypical probiotic food” [14]. 

At present, several studies support the idea that HM harbours a complex microbial community, which is a source of a huge number of viable commensal, mutualistic, or potentially probiotic bacteria for developing infants and their gut (estimated as 1 × 10^5^ to 1 × 10^7^ bacteria daily for an infant receiving 800 mL/day of HM) [15]. 

The first characterizations of HMM in healthy women were based on culture-dependent techniques [16,17], whose limit was to allow the isolation of only a limited number of bacterial genera: studies performed using culture-dependent methods revealed that facultative anaerobic or prevalently aerobic species were the main colonizers of the HM ecosystem, with *Streptococcus* and *Staphylococcus* being the most frequently isolated and abundant bacterial groups, together with skin-derived or environmental contaminants (i.e., *Propionibacterium* and genera belonging to the Enterobacteriaceae family). However, well-known intestinal probiotic bacteria (i.e., *Bifidobacterium* and *Lactobacillus*) had been isolated as well.

The development of culture-independent techniques, such as quantitative polymerase chain reaction (qPCR) and, later on, next generation sequencing (NGS), mostly based on 16S rRNA gene, has allowed the characterization of the composition and diversity of HMM in deep detail, and has documented a huge number of bacteria in breast milk and a high variability in HMM composition. Indeed, microbiome research has experienced an unprecedented rate of data productivity in the last decade. While allowing for an everyday-increasing amount of evidences on microbiome–human dynamics and interactions, such an amount of studies and results have also highlighted considerable limitations in data obtained from the molecular approaches. For instance, NGS studies provide only a sense of relative abundance because actual bacterial counting is lost during amplification. Another limitation of molecular methods is the so called “viability bias”, i.e., the detection of bacterial DNA does not mean the corresponding microorganism is alive in the original sample. Moreover, different methods of DNA extraction and processing are known to have an impact on the results and their reproducibility [18]. Nonetheless, the amount of molecular data available today on HM composition constitutes good evidence that HM is not only consistently present in healthy lactating women but is also likely to play an ecological role in mother and infant health. 

Fitzstevens et al. [19] recently performed a systematic review including twelve studies which used culture-independent methods to identify bacteria at the genus level in HM from healthy women. In most of these studies, *Streptococcus* and *Staphylococcus* appeared to be the predominant genera in HM independently from the geographic location of the study and from the selected technique (qPCR or NGS). Culture independent studies have also allowed the detection in HM of obligate anaerobic, gut-associated genera, such as *Bacteroides*, *Blautia*, *Dorea*, and *Faecalibacterium* [20]. Single studies previously performed had suggested the existence of two different “core HMM”, consisting of seven (*Staphylococcus*, *Streptococcus*, *Bacteroides*, *Faecalibacterium*, *Ruminococcus*, *Lactobacillus*, and *Propionibacterium* [21]) or nine (*Staphylococcus*, *Streptococcus*, *Serratia*, *Pseudomonas*, *Corynebacterium*, *Ralstonia*, *Propionibacterium*, *Sphingomonas*, and uncultured members of *Bradyrhizobiaceae*) microbial genera [22]. *Staphylococcus*, *Streptococcus*, and *Propionibacterium* were the only three genera reported as predominant in both these studies, suggesting that the concept of “core microbiota” was probably dependent on the geographic location of the study, and on the method used for HM collection, storage, and analysis. The predominance of *Streptococcus* and *Staphylococcus* genera in HM was confirmed also in the study by Lackey et al. [23], who analysed HM and neonatal faecal samples from 11 geographical sites through sequencing of the V1-V3 region of the bacterial 16S rRNA gene. This study highlighted that HMM composition and diversity are characterized by huge intra- and inter-population variability. In this context, significant associations between individual bacterial genera in HM and infants’ faeces were hard to detect; however, strong, even if geographically specific, associations between the complex microbial communities of infant’s faeces and MOM could be reported by multivariate analyses. 

The very recent work by Togo et al. [24] summarized the results of 242 papers from 38 countries, including data from over 15,000 samples. Data from breast tissue, colostrum, and HM samples were analysed at the species level and more than 800 bacterial species, mainly belonging to Proteobacteria and Firmicutes, were identified. The most frequently detected bacterial species were *Staphylococcus aureus*, *Staphylococcus epidermidis*, *Streptococcus agalactiae*, *Propionibacterium acnes*, *Enterococcus faecalis*, *Bifidobacterium breve*, *Escherichia coli*, *Streptococcus sanguinis*, *Lactobacillus gasseri*, and *Salmonella enterica*. The breast and HM microbiota shared 49% of the species of their repertoire with the gut, 30% with the vagina, 28% with the urinary tract, 28% with the respiratory tract, and 21% with the oral cavity. Approximately 300 bacterial species were found only in the breast and HM microbiota and not in other human microbial niches, suggesting the existence of a breast and HM microbiota with distinctive features.

Variation in HMM composition among women and populations could be related to several factors, including but not limited to length of gestation, delivery mode, time postpartum, and maternal factors such as diet, intake of specific nutrients (i.e., HMOs), and use of antibiotics/probiotics. In addition, it is unclear how much of the geographical variability in HMM composition is truly related to actual differences among studied populations or rather to differences in the setting and procedure of milk collection, storage, and analysis. However, it should be noted that recent large-scale studies have documented that infants’ GM and their mothers’ HMM are more similar within than across cohorts, suggesting that the characteristics of both GM and HMM could be tailored not only to the infants’ and mothers’ lifestyle but also to specific environmental settings [23]. 

## 3. Origin and Determinants of the Human Milk Microbiome

The origin of bacteria in HM is not well established, but growing literature suggests that the genesis of HMM is a complex phenomenon. Two different routes, which are probably not mutually exclusive, have been proposed for HMM establishment: microbes can colonise HM through surface skin contamination and retrograde flow (salivary backwash) during breastfeeding or, alternatively, they might translocate to HM through a more speculative gut-mammary route, the so-called “entero-mammary pathway”.

Biagi et al. examined HMM, infant’s oral as well as gut microbiota in a cohort of healthy term infants and their mothers. According to their data, the infant’s mouth, which is the transition point for HM to reach the gut, could play a relevant role in shaping the characteristics of both HMM and the infant’s gut microbiota [25]. The existence of a salivary backwash is also supported by data from the Canadian Healthy Infant Longitudinal Development (CHILD) Study: in a subgroup of mother–infant dyads from the CHILD cohort, the determinants of HMM composition and diversity were examined. The mode of HM feeding (direct breastfeeding vs. provision of MOM expressed through a breast pump) was found to be associated with HMM distinctive features. Specifically, expressed MOM had a higher content of potential pathogens and a lower abundance of bifidobacteria compared to HM provided directly from the breast [26]. This observation supports a retrograde inoculation hypothesis, with the infant’s saliva seeding HMM during breastfeeding. In addition, Ruiz et al. examined the microbiota from colostrum expressed by pregnant women before delivery and from the mouth of those women’s infants. Several taxa were shared by the two ecosystems, with *Streptococcus* and *Staphylococcus* being the most abundant [27]. These observations document the presence in human colostrum, before delivery, of bacteria which are typical of the infants’ mouth; this supports the hypothesis of a direct colonization of the infant’s mouth through breastfeeding, as part of a dynamic cycle with a bidirectional flow of bacteria between the mother and the infant during suckling. In a prospective study, Biagi et al. recruited a cohort of HM-fed moderately preterm infants (gestational age 32–34 weeks) and examined their mother’s milk microbiota before and after the beginning of actual breastfeeding [28]. The infant’s latching to the mother’s breast produced an increase in HMM diversity and a shift in its composition, characterised by the dominance of typical oral microbes, such as *Streptococcus* and *Rothia*. The changes in HMM following the beginning of breastfeeding were associated with an increase in the abundance of beneficial bacteria, such as *Bifidobacterium,* in the infants’ faeces and to a reduction of *Pseudomonas* in oral samples. 

On the other side, according to the entero-mammary pathway theory [29], maternal intestinal bacteria would be able to reach extraintestinal sites. During late pregnancy and lactation, these bacteria would first translocate through the intact maternal gut mucosa by internalization in the dendritic and CD18+ cells, and then circulate to the mammary gland via the lymphatic and blood circulation [30]. The existence of an endogenous route for the colonization of the mammary gland by gut bacteria from the mother could be supported by the retrieval, in HMM, of DNA from anaerobic bacterial families, such as Lachnospiraceae, Ruminococcaceae, and Bacteroidaceae, which are commonly found in the adult human intestine [25]. Furthermore, Ruiz et al. recently reported the presence of typical oral bacteria in precolostrum samples, collected during late gestation and thus before any contact of the infant’s mouth to the breast [27]. The retrieval of these bacteria in precolostrum confirms that at least some bacteria are present in HM before delivery and before the beginning of breastfeeding; however, at present an actual mechanism for an entero-mammary bacterial translocation has yet to be demonstrated. Alternatively, the endogenous route could concern only bacterial antigens, such as DNA or proteins, instead of living bacterial cells.

Putting these observations together, HMM may be considered as a dynamic crossroad of different and inter-related bacterial communities. However, HMM assembly is far from clear and mother–infant microbial dynamics during breastfeeding need further investigation because of the critical role played by the microbial colonization of the infant’s gut in immune system education and development.

In the complex scenario of the HMM assembly, it is likely that also maternal (BMI, parity, and mode of delivery), infant (gestational age, gender), and environmental factors (geographic study location, substance exposure) interact in influencing HMM composition. 

Several studies have explored environmental determinants of microbial composition and diversity in HMM, among which geographical and lifestyles differences have been shown to explain a certain degree of interindividual variation [23,31,32]. In the study by Kumar et al., HM samples were collected from selected populations in Europe, Africa, and Asia, and analysed for fatty acid profile and microbiota features [31]. In addition to differences related to mode of delivery, both fatty acid and microbiota composition were related to the geographical study location. Similarly, Gomez-Gallego et al. examined the influence of mode of delivery and geographic location on HMM and HM metabolites, evaluated by nuclear magnetic resonance: a high variability in HM metabolites was documented across study sites, and variations in HM metabolome were found to be associated with specific features of HMM [32]. These observations were confirmed by the study by Lackey et al. [23], where HM and infant gut microbiota were examined across multiple geographical sites across the world. Even if a limited number of core genera both in HM and infant’s faeces were shared among different populations, a significant variability was documented within and across cohorts. 

Other environmental factors that could shape HMM are exposure to disinfection agents [33], exposure to maternal and neonatal antibiotics [34], and the structure of human social networks. A study performed in Central Africa Republic within a small-scale society documented a relationship between HMM composition and diversity and the size of the mother–infant social network and the amount of care received by the infant [35].

Other factors associated with an overall variation in the HMM structure include mode of delivery [26,32,34,36] and lactational stage [37,38]. Differences in the HMM structure between women who delivered vaginally and those who delivered by C-section have been frequently reported, though the specific taxa involved (*Lactobacillus* spp., *Bifidobacterium* spp.) vary among cohorts and studies [26,32,34,36]. In general, HMM from mothers who delivered vaginally is richer and more diverse compared to HMM from mothers who underwent a C-section [36]. Differences were also documented in HMM between mothers who had received antibiotics in the peripartum compared to those who had not [34], and between elective vs. non-elective C-section [37].

Differences in the composition of HMM among colostrum, transition, and mature milk have been reported by some authors, with an increased abundance of typical oral inhabitants in transition and mature milk, and higher counts of *Bifidobacterium* at later stages of lactation, though such differences have not been consistently observed across studies [37,38,39].

The influence of length of gestation has also been investigated, with higher counts of *Bifidobacterium* spp. in milk samples from term-delivering compared to preterm-delivering mothers. As for the degree of premature delivery, a progressive increase in total bacterial count appears to be associated with increasing length of gestation [38]. In the two studies by Biagi et al. [25,28], examining two distinct cohorts of term-delivering vs. moderately preterm-delivering mothers from the same study site, the differences in HMM were significant, especially when HM samples collected in the first few days after delivery from mothers of preterm infants were compared to those from mothers of term infants. Nonetheless, differences between the two cohorts appeared to decrease when moderately preterm infants started to be fed directly at their mothers’ breast, strengthening the hypothesis of a direct influence of breastfeeding on HMM composition. 

It is likely that milk components other than HMM, such as HMOs, milk fatty acids, hormones, immune cells, and antibodies, could modulate the milk ‘‘microenvironment’’, possibly affecting the composition of its microbial community [5,32,40]. 

Although HMM studies have mainly focused on bacteria, HM harbours and may also vehiculate yeasts or viruses. Pannaraj et al. examined the relationship in the virome composition between HM and infant stools: the milk virome showed a higher diversity compared to the infant gut virome, and both showed distinctive features compared to the adult sites’ virome. Although differences in viral microbiota composition between HM and stools were substantial, a significant number of viruses was shared between the two ecosystems, with a large proportion of bacteriophages transmitted from the mother to the infant through breastfeeding, which could contribute actively at modifying HMM bacterial composition [41]. To date, the features of HM’s eucariome have not been explored yet. 

## 4. Human Milk Microbiome and Its Influence on Infant Gut Colonisation and Health

Human breast milk is considered the gold standard for infant nutrition. Beyond its nutritional benefits, breastfeeding is known to reduce respiratory and gastrointestinal infections in early life and decrease the risk of non-communicable diseases such as atopy, diabetes, obesity, and inflammatory bowel disease. As for preterm infants, exclusive HM feeding is also associated with improved neurodevelopmental outcomes and reduced risk of NEC [42,43,44]. 

Among bioactive factors, which could modulate clinical outcomes, HMOs and HMM appear to exert a synergic action in shaping the infant’s GM [5,13]. HMOs act as prebiotics promoting the proliferation of specific bacteria, including *Bifidobacterium* [45]—the most important probiotic group residing in the gut of healthy term infants [8]. Microbes in MOM seed the infant’s oral cavity and gut, providing pioneer colonizer bacteria and enrichment of bacteria associated with beneficial effects, and contributing to the establishment of the infant intestinal and oral microbiome [46,47]. Indeed, multiple studies have documented that breast milk and term infants’ faeces share specific microbial strains of *Bifidobacterium, Lactobacillus, Enterococcus*, and *Staphylococcus* [48,49,50]. It has also been shown that GM of breastfed infants differ from non-breastfed individuals and that early fingerprints may persist into adulthood. GM plays crucial roles in early life metabolic and immune system homeostasis and development [51,52]: host-microbiota interplay promotes barrier function, mucosal, and systemic immune function, leading from a Th2-biased toward a balanced Th1/Th2 immune response [53]. It also “educates” the gut-associated lymphoid tissue, allowing the establishment of a tolerant state between the microbiota and the immune system [54]. Furthermore, patterns of initial colonisation affect host metabolic function, including fat deposition, circulating leptin levels, and insulin resistance in the neonatal period [8]. In early life, being a term, vaginally delivered, and exclusively breastfed infant is considered the ideal condition for developing a healthy microbiota. However, gut colonization occurs differently in preterm infants [55]. Preterm gut structural and immunological immaturity together with a unique set of environmental conditions (mode of birth, antibiotic administration, setting of care, and nutritional exposure) contribute to abnormal bacterial colonisation and decreased microbial diversity compared to term infants. Such dysbiosis may result in an inflammatory response exacerbated by an immature innate immune response that increases the risk of diseases such as NEC and late onset sepsis (LOS), both significant causes of mortality. Many episodes of LOS are due to gut-derived organisms, and changes in the intestinal barrier contribute to both LOS and NEC [56]. NEC does not have a specific microbial signature, however large-scale variation in bacterial taxa at high phylogenetic level (i.e., differential abundances in phyla Proteobacteria, Firmicutes, and Bacteroidetes) was reported to precede NEC [57,58]. The increase in proteobacteria, along with increased enterocyte Toll like receptor 4 activity in neonates with NEC, suggests a hyperinflammatory response to a dysbiotic microbiome [59,60]. A recent paper by Fundora et al. aimed at assessing the causality for intestinal dysbiosis leading to NEC: Although causality was supported by biological plausibility of an aetiological role of Gram-negative bacteria and temporal evidence of enteric dysbiosis preceding NEC, consistency among studies was low, and the effect appeared to be non-specific [61].

Beyond the unique set of clinical factors that predispose preterm infants to gut dysbiosis, infant feeding has a huge impact in shaping the GM. Several studies have documented substantial differences in the composition of GM for preterm infants fed MOM, donor HM (DHM), and formula, with MOM leading to the highest microbial diversity compared to both DHM and formula [62], as well as a more gradual acquisition of diversity compared to formula [63]. In addition, provision of MOM appears to attenuate the detrimental effect of low birth weight/low gestational age, leading to an ordered succession of bacterial phenotypes independently from the infant’s degree of immaturity [63]. Recent data also suggest that variation in GM bacterial diversity and composition induced by different feeding practices could be associated with the extent of systemic oxidative stress, measured as levels of urinary F2-isoprostane metabolites in very low birth weight preterm infants [64].

Thus, it can be speculated that both HM and HMM may act as early mediators between the development of GM in early life and health outcomes. *Lactobacillus* and *Bifidobacterium* species isolated from HM appear to play a beneficial role in the infant’s health [52]. These bacteria have been extensively studied, given the potential of many strains belonging to *Lactobacillus* or *Bifidobacterium* spp. to be used as probiotics. Some *Lactobacillus* and *Bifidobacterium* strains [65] have demonstrated an antimicrobial activity and a role in the development of the gut barrier function, protection against infectious diseases, metabolism, immunomodulation, and neuromodulation in studies in vitro and in vivo, both in animal models and in human clinical trials. Moreover, the frequent retrieval of *Streptococcus* and *Staphylococcus* spp. in the faeces of breast-fed infants, as well as in their mothers’ milk microbiota, might call for a possible biological role for these bacteria during the infant’s microbiota assembly [28]. 

Beyond the seeding effect on infant gut of HM beneficial bacteria, the presence of bacterial DNA, including DNA derived from dead bacterial cells, may also play a role in the infant immune development. Ward et al. [66] analysed HM through a metagenomic approach, identifying abundant and prevalent functions in HMM compared to the infant GM, and described putative immunomodulatory motifs in microbiota-derived DNA sequences. Among them, unmethylated cytosine phosphate guanine (CpG) dinucleotides within bacterial DNA are known as strong immune stimulators, acting through toll-like receptor 9 [67]. Furthermore, immunosuppressive DNA sequences that can counteract the effects of CpGs have been discovered in commensal *Lactobacillus* [68]. Therefore, it is likely that immune-modulatory motifs in HM-derived DNA could contribute to the preterm infants’ immune balance by decreasing the exaggerated inflammatory response to colonizing bacteria, which is involved in the pathogenesis of NEC. 

## 5. Human Milk Microbiota: Implications for Preterm Infants’ Care and Role of Donor Milk

Exclusive HM feeding is of utmost importance for preterm infants, as MOM-feeding is linked to a reduction in the incidence of life-threatening diseases, such as NEC and LOS, as well as to an improvement in preterm infants’ neurodevelopment [69]. HM feeding promotes a healthy microbiome, supports appropriate maturation of the developing immune system [70], and protects infants against infections mainly via secretory IgA antibodies, as well as via immunomodulatory factors such as enzymes (lysozyme, lactoferrin, etc.), cytokines, complement system components, leukocytes, oligosaccharides, nucleotides, lipids, and hormones, which ensure host defence against infections and modulate the immune response [3].

The beneficial role of HM in infants’ feeding becomes even more crucial for preterm infants born with a very low birth weight [71], whose structural immaturity conflicts with the need to sustain a huge growth and maturation after birth. Preterm birth exposes the infants to significant risk of severe infections in early life, and this is associated with adverse outcomes, including death, chronic lung disease, and neurodevelopmental impairment [72].

However, providing an exclusive HM diet to preterm infants presents a variety of challenges related to prematurity itself and to hospitalization. For preterm infants, the term “exclusive HM feeding” covers a range of feeding practices other than direct breastfeeding, such as the use of fresh vs. frozen expressed breast milk given by bottle or tube feeding, the addition of HM fortifiers, and a variable duration of exclusive HM feeding. Some of these interventions (pumping, storage process, etc.) might affect the nutritional and non-nutritional components of HM. The results of the CHILD study reported a reduced risk of wheezing, asthma, and high BMI among breastfed children [73]. These associations were stronger among infants fed directly at the breast compared to those receiving expressed breast milk, although both practices were superior to infant formula. Furthermore, Biagi et al. [28] showed in a cohort of moderately preterm infants that HMM composition changed following the infant’s latching to the mother’s breast, shifting towards a more diverse microbial community dominated by typical oral microbes, with a high abundance of *Bifidobacterium* in the infants’ faeces collected after actual breastfeeding had started. Taken together, these evidences suggest that latching, the act of suckling and/or skin-to-skin contact, may contribute to the protective effect of breastfeeding. Specifically, the infant’s latching to the mother’s breast might constitute an independent factor helping the health-promoting assembly of the infant gut microbiome, and thus an early initiation of breastfeeding should be encouraged and promoted among mothers of preterm infants.

When MOM is unavailable or insufficient, the latest recommendations identify DHM, provided by a qualified HM bank, as the optimal alternative or supplement to MOM [74]. In order to inactivate viral and bacterial agents, current international guidelines recommend HM pasteurization using the Holder method [75], which guarantees DHM microbiological safety but is also known to impair several nutritional and biological HM properties [76,77]. Furthermore, it is likely that pasteurization, while inactivating potentially harmful microorganisms, might interfere with the resident HMM, thus impairing the probiotic effect of MOM. A recent study demonstrated that MOM-fed preterm infants showed different microbial patterns in their GM compared to DHM-fed infants [78], with a significantly higher abundance of Bifidobacteriaceae and lower Staphylococcaceae, Clostridiaceae, and Pasteurellaceae. The gut microbial profile in formula-fed infants was different to that of infants receiving MOM, while infants fed DHM showed a microbial profile more like infants fed their mothers’ milk.

Even if some analogies are documented between DHM and MOM in terms of the composition of GM in preterm infants, little is known about the specific effect of DHM on preterm GM and its potential functional implications. Some authors postulate that preterm infants fed DHM or formula are devoid of contact with HMM [79], and for this reason strategies which allow an early exposure of preterm infants to the HMM have been proposed. To this purpose, Cacho et al. [80] inoculated DHM with small amounts of MOM from mothers of preterm infants. According to viable cell counts and microbiome analysis, they suggested that DHM incubated with 10% MOM for 4 h was a reasonable restoration strategy. Alternatively, it has been proposed that, when MOM is not available, a detailed screening of HM donors, including HM routine assay for cytomegalovirus, would permit to avoid the HM pasteurization in a certain proportion of donors [81]. Furthermore, when only pasteurized DHM or formula are available, their supplementation with probiotic strains isolated from HM might partially cover for the missing HMM function [79]. 

No specific evaluation of potential probiotic properties of DHM in its current form is available at present. It appears reasonable that, given the pasteurization process underwent by DHM, no viable beneficial bacteria would be retrieved in pasteurized DHM. 

However, according to recent evidence, the probiotic effect of beneficial microbes does not rely necessarily on their viability, but their interaction with the host might be based on the capacity of human cells to recognise specific bacterial components or products, giving rise to responses that commonly involve the mucosa-associated lymphoid tissue and, therefore, the immune system. For this reason, the term “para-probiotics” or “ghost probiotics” has been proposed for “non-viable (more often heat-inactivated) microbial cells (intact or broken) or crude cell extracts (i.e., nucleic acids, cell-wall components), which, when administered (orally or topically) in adequate amounts, confer a benefit on the human or animal consumer”. Ghost probiotics might act through several mechanisms, including the modulation of various steps of the inflammatory and immune responses; given their potentially higher safety compared to standard viable microbial cells, a potential application for para-probiotics has been recently suggested also for preterm infants [82].

In analogy with ghost probiotics (beneficial microbial cells inactivated by heat treatment), one can speculate that DHM processed through Holder Pasteurization (HoP), despite not retaining intact microbial cells, might harbour a ghost microbiota, whose clinical significance has not been yet investigated. The exact role of this putative “ghost microbiota” in DHM still needs to be described.

## 6. Conclusions and Future Directions

Further studies are required to evaluate in detail the characteristics and biological role of HMM. It is of paramount importance to first understand what constitutes a healthy reference for HMM, as well as what are the determinants of milk microbial community structure. Such increased knowledge would shed light on how this important source of microbes impacts on infant health, and how the process might be positively influenced by clinical practice in preterm infants. More targeted studies are warranted to define the mechanism by which the HMM impacts immune and gut development in the infant host. Indeed, it appears that this field of research, merging microbial ecology and clinical practice, has reached a point in which observational studies should be gradually replaced by mechanistic approaches, which would be able to provide a finer comprehension on microbes–mother–infant interaction during this crucial period of human life. Knowledge that goes beyond the community profile and starts to provide functional insights into HMM, as well as other non-viable probiotic components of HM, may provide new opportunities to improve health or modify disease outcomes. 

## Abbreviations

DHM	donor human milk
DOHaD	developmental origin of health and disease
GM	gut microbiota
HM	human milk
HMM	human milk microbiota
HMO	HM oligosaccharides
HoP	Holder Pasteurization
LOS	late-onset sepsis
MOM	mother’s own milk
NEC	necrotizing enterocolitis
NGS	next generation sequencing
qPCR	quantitative polymerase chain reaction
rRNA	ribosomal RNA
